# Dr. Hooper's Physician's Vade-Mecum; or Manual of the Principles and Practice of Physic. New Edition, Considerably Enlarged and Improved, with an Outline of General Pathology and Therapeutics

**Published:** 1842-07

**Authors:** 


Art. V.—*Dr. Hooper's Physician's Vade Mecum; or Manual of the Principles and Practice of Physic. New Edition, considerably Enlarged and Improved, with an Outline of General Pathology and Therapeutics.* By W. A. Guy, m.b. Cantab., Professor of Forensic Medicine, King's College, London; and Physician to the King's College Hospital.—*London,* 1842. Sm. 8vo, pp. 493.

It is often useful for the practitioner as well as the student to have a concise and correct epitome of the history and treatment of diseases to refer to, either as an awakener of knowledge laid by in the mind but perhaps forgotten for the time, or as a suggester of new views and plans for further investigation. Dr. Hooper's well-known Vade Mecum has long served this purpose; and to enable it to continue to do so, it was necessary to alter what was antiquated and obsolete, in its pages in conformity with recent improvements and newer doctrines. This task has been excellently well executed by Dr. Guy; and the student and general practitioner may confidently turn to the present edition for the information which it is the scope of the work to supply. We rather think, however, judging from the character of the alterations made in the work by Dr. Guy, that a less philosophical editor might have produced a work more suited to the wants of those likely to consult it. We think that the admirable First Part, extending to more than one third of the whole book, entirely contributed by Dr. Guy, and devoted to the subjects of medical study, physiology, general pathology, and therapeutics, is rather out of place in a work of this kind, which, in our opinion, should be confined to a concise exposition of the history and treatment of special diseases. By omitting this first or general Part, and devoting the whole attention to correct, amend, and especially to condense the Second Part, the editor might have presented us with a work of one half the size and price, and no less calculated than the present to fulfil what we conceive to be the true scope of a vade mecum. If, by such a plan, however, we had been altogether deprived of the matter contained in the first part of the present volume, we should have regretted its adoption. But we do not consider this as at all a necessary consequence. On the contrary, we think Dr. Guy instead of giving us one book should have given us two, namely, a treatise on general pathology and therapeutics, and an epitome of practical medicine; and when a new edition of the present work is called for, we strongly advise this course to be pursued; the vade mecum being condensed to a five-shilling duodecimo, and the new treatise expanded, if such is the author's pleasure, into a ten-shilling octavo.

